# The Complete Chloroplast Genome Sequences of Three Veroniceae Species (Plantaginaceae): Comparative Analysis and Highly Divergent Regions

**DOI:** 10.3389/fpls.2016.00355

**Published:** 2016-03-23

**Authors:** Kyoung Su Choi, Myong Gi Chung, SeonJoo Park

**Affiliations:** ^1^Department of Life Sciences, Yeungnam UniversityGyeongsan, South Korea; ^2^Department of Biology, The Research Institute of Natural Science, Gyeongsang National UniversityJinju, South Korea

**Keywords:** veroniceae, *veronica*, chloroplast genome, phylogenetic tree, divergent regions

## Abstract

Previous studies of *Veronica* and related genera were weakly supported by molecular and paraphyletic taxa. Here, we report the complete chloroplast genome sequence of *Veronica nakaiana* and the related species *Veronica persica* and *Veronicastrum sibiricum.* The chloroplast genome length of *V. nakaiana, V. persica*, and *V. sibiricum* ranged from 150,198 bp to 152,930 bp. A total of 112 genes comprising 79 protein coding genes, 29 tRNA genes, and 4 rRNA genes were observed in three chloroplast genomes. The total number of SSRs was 48, 51, and 53 in *V. nakaiana, V. persica*, and *V. sibiricum*, respectively. Two SSRs (10 bp of AT and 12 bp of AATA) were observed in the same regions (*rpoC2* and *ndhD*) in three chloroplast genomes. A comparison of coding genes and non-coding regions between *V. nakaiana* and *V. persica* revealed divergent sites, with the greatest variation occurring *petD*-*rpoA* region. The complete chloroplast genome sequence information regarding the three Veroniceae will be helpful for elucidating Veroniceae phylogenetic relationships.

## Introduction

Chloroplast (cp) are photosynthetic organelles that provide energy to green plants (Douglas, 1990). Chloroplast genomes are valuable sources of phylogenetic information because of their relatively stable genome structure and higher evolutionary rate relative to mitochondrial genomes. Chloroplast genomes consist of a large inverted repeat (IR) separated by a large single-copy (LSC) region and a small single-copy (SSC) region. Approximately 100–130 genes are located along the circular genome structure of chloroplasts. These genes exhibit a highly conserved gene order and contests (Wicke et al., 2011) and typically encode ~79 proteins, ~30 transfer RNAs and four ribosomal RNAs. However, some parasitic plants contain fewer genes than photosynthetic plants (Funk et al., 2007; Wicke et al., 2013). Cp genome sequences are useful genetic markers for DNA barcoding, transplastomic studies and evolutionary studies from the population level, as well as for phylogenetic relationships (Bock and Khan, 2004; Jansen et al., 2007).

The genus *Veronica* is a member of the family Plantaginaceae in the order Lamiales, which consists of 24 families and ~23,000 species (Angiosperm Phylogeny Group, 2009). Almost all families of Lamiales are included in Scrophulariaceae. However, molecular analysis of Scrophulariaceae was only recently conducted and reclassification (Olmstead and Reeves, 1995; Albach et al., 2005). *Veronica* were traditionally circumscribed to Scrophulariaceae. However, molecular studies conducted by Olmstead et al. (2001) separated several families, including Veroniceae (Plantaginaceae). The tribe Veroniceae was first established in 1828 by Duby to include the genera *Bseeya, Botryopleuron, Calorhabdos, Hebe, Lagotis, Paederota, Picrorhiza, Synthris, Wulfenia, Veronicastrum*, and *Veronica*. Subsequent molecular studies discussed the tribe Veroniceae and proposed a revised classification for the genus *Veronica*. Albach and Chase (2001) and Albach and Meudt (2010) included *Veronica, Hebe, Beccabunga, Synthyris, Cochlidiosperma, Pellidosperma, Chamaedrys, Stenocarpon, Pocilla, Pentasepalae*, and *Pseudolysimachium* into a genus *Veronica.*

DNA barcoding can be used to elucidate plant relationships at the species level. Ribosomal ITS sequences (Alvarez and Wendel, 2003) and non-coding region *trnL* intron (Taberlet et al., 2006) and *psbA*-*trnH* regions (Kress et al., 2005) are useful markers for identifying plant specimens and relationships. Previous phylogenetic analyses of Veroniceae used markers of the nuclear DNA ITS and CYC2 and plastid DNA *rbcL, psbA*-*trnH, trnL*-*trnL*-*trnF, rps16* introns, and *rpoB*-*trnC* (Albach and Chase, 2001; Wagstaff et al., 2002; Albach et al., 2004a,b, 2005; Albach and Meudt, 2010). However, the relationship among the subgenus in *Veronica* (including subgenus *Pseudolysimachium*) was paraphyletic and not well supported (Albach et al., 2004a,b; Albach and Meudt, 2010).

Here, we report the first chloroplast genome of three Veroniceae (*Veronica nakaiana*, subgenus *Pseudolysimachium*; *Veronica persica*, subgenus *Pocilla*; *Veronicastrim sibiricum*). The specific goals of the present study were to: (1) present the complete chloroplast genome sequence of three Veroniceae, (2) evaluate the phylogenetic position of 78 coding genes in three Veroniceae plants, (3) compare the coding and non-coding regions of three Veroniceae plants and suggest effective regions of the chloroplast genome for phylogenic analyses in Veroniceae.

## Materials and methods

### Plant samples and sequencing

This research was approved by the Korean National Arboretum. *V. nakaiana, V. persica*, and *V. sibiricum* were obtained from the living collections at the greenhouse of Yeungnam University. Total DNA was extracted using a DNeasy Plant Mini Kit (Qiagen Inc., Valencia, CA, USA) and quantified using a HiGenTM Gel and PCR Purification System (Biofact Inc., Daejeon, Korea). Genomic DNA was sequenced using Illumina Miseq (Illumina Inc., San Diego, CA). *V. nakaiana, V. persica*, and *V. sibiricum* were sequenced to produce 3,205,654–4,475,346 raw reads and 301 bp were obtained from them. These paired-end reads were aligned with *Sesamum indicum* (JN637766.2), and 207,913 to 1,450,476 reads were mapped to the reference genomes. The fold coverage of *V. nakaiana, V. persica*, and *V. sibiricum* was 1223, 1911, and 404, respectively. The genome coverage was estimated using the CLC Genomics Workbench v7.0.4 software (CLC-bio, Aarhus, Denmark).

### Annotation, genome mapping, and sequence analysis

DOGMA (Wyman et al., 2004) was used to annotate the *V. nakaiana, V. persica*, and *V. sibiricum*. Initial annotation, putative starts, stops, and intron positions were determined by comparison with homologous genes in other cp genomes. tRNA genes were annotated using DOGMA and tRNAscan-SE (Schattner et al., 2005). To compare the structure and gene content within the three Veroniceae, plants were aligned using MAFFT (Katoh et al., 2002). A circular cp genome map was drawn using the OGDRAW program (Lohse et al., 2007).

### Repeat structure

REPuter was used to visualize both forward, palindrome, reverse and complement sequences, with a minimum repeat size of 30 bp and a sequence identity greater than 90% (Kurtz and Schleiermacher, 1999). The simple sequence repeats (SSRs) in the three Veroniceae plant cp genomes were detected using Phobos v. 3.3.12 (http://www.ruhr-uni-bochum.de/ecoevo/cm/cm_phobos.htm). Repeats were ≥10 sequence length and three repeat units for mono-, di-, tri-, tetra-, penta-, and hexanucleotides.

### Phylogenetic analysis

A total of 78 coding genes of 18 Lamiles plants were compiled into a single file of 71,314 bp and aligned with MAFFT (Katoh et al., 2002). Seventeen Lamiales (including *V. nakaiana, V. persica*, and *V. sibiricum*) were selected as the in-groups and *Buxus* was included as the outgroup (Supplementary Table 1). Maximum likelihood (ML) analyses were performed using RAxML v7.4.2 with 1000 bootstrap replicates and the GTR+I+G model (Stamatakis, 2006).

### Divergence hotspot identification

The three completed chloroplast genome sequences (*V. nakaiana, V. persica*, and *V. sibiricum*) were aligned using MAFFT (Katoh et al., 2002). To analyze nucleotide diversity (Pi), the total number of mutations (Eta) and average number of nucleotide differences (K) were determined using DnaSP (Librado and Rozas, 2009).

## Result

### Genome organization of three veroniceae

**Figure 1:**
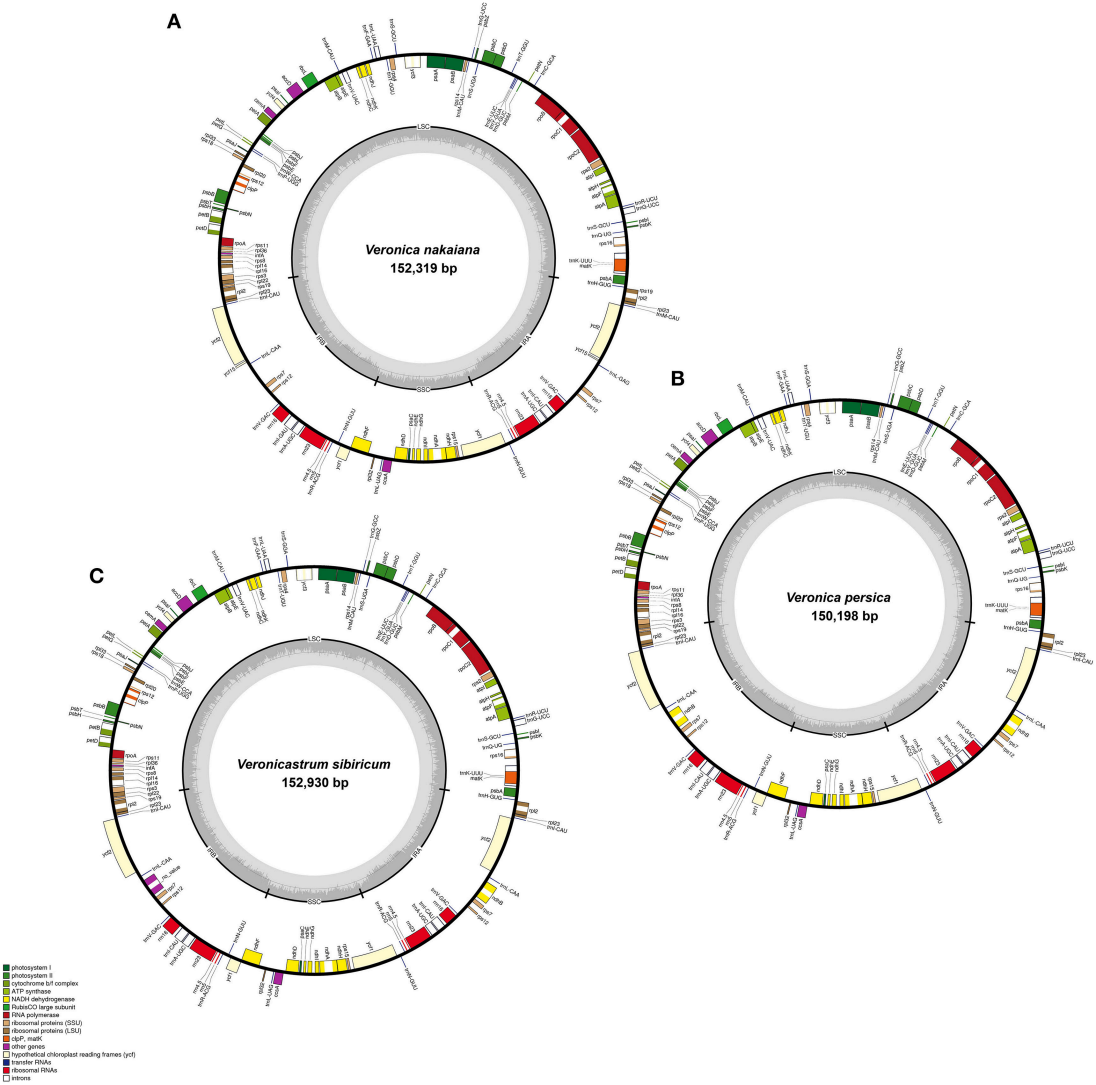
**Chloroplast genome of *Veronica nakaiana*, *Veronica persica*, and *Veronicastrum sibiricum***. Genes inside the circle are transcribed clockwise, gene outside are transcribed counter-clockwise. The dark gray inner circle corresponds to the GC content, the light-gray to the AT content. **(A)** *Veronica nakaiana* chloroplast genome, **(B)** *Veronica persica* chloroplast genome, **(C)** *Veronicastrum sibiricum* chloroplast genome.

The total genome size of the Veroniceae (Figure 1) is 152,319 bp in *V. nakaiana* (Genebank accession no. KT633216), 150,198 bp in *V. persica* (Genebank accession no. KT724052), and 152,930 bp in *V. sibiricum* (GeneBank accession no. KT724053). Chloroplast genomes displayed a typical quadripartite structure, consisting of a pair of IRs (25,465–25,757 bp) separated by the LSC (81,850–83,616 bp) and SSC (17,418–17,800 bp) regions (Figure 1, Table 1). Among the tree species, the cp genomes (LSC, SSC, and IR regions) of *V. sibiricum* are larger than that of the other species, and *V. persica* is smallest (Table 1). Both contain 112 different genes, including 79 proteins (nine large ribosomal subunits, 12 small ribosomal subunits, four DNA-dependent RNA polymerases, one translation initiation factor, 45 genes encoding photosynthesis-related proteins and eight genes encoding other proteins), 29 tRNA genes and 4 rRNA genes were annotated (Figure 1, Table 1) in three Veroniceae chloroplast genomes.

**Table 1:** **Comparison of features of *Veronica nakaiana, V. persica*, and *Veronicastrum sibiricum***.

**Feature**	***Veronicax nakaiana***	***Veronica persica***	***Veronicastrum sibiricum***
Genome size	152,319	150,198	152,930
Large single copy	83,195	81,850	83,616
Small single copy	17,702	17,418	17,800
Inverted repeat	25,711	25,465	25,757
**A-T (%)**			
Total genome	62.1%	62.1%	61.7%
LSC	64%	64%	63.5%
SSC	68.3%	62.4%	67.7%
IR	56.8%	56.8%	56.7%
Number of genes	79	79	79
Number of tRNAs	29	29	29
Number of rRNAs	4	4	4
**SEQUENCING INFORMATION (PAIR-END READS)**			
Raw data read number	5,430,065	8,950,692	8,174,506
Mapped read number	707,631	1,106,738	207,913
Chloroplast coverage (x)	1223	1911	404

The overall AT content was 61.7–62.1%, indicating nearly identical levels among the three complete Veroniceae cp genomes. The AT content was 63.5–64, 62.4–68.3, and 56–56.8% of the genome for the LSC, SSC, and IR regions, respectively (Table 1).

### IR expansion and contraction

**Figure 2:**
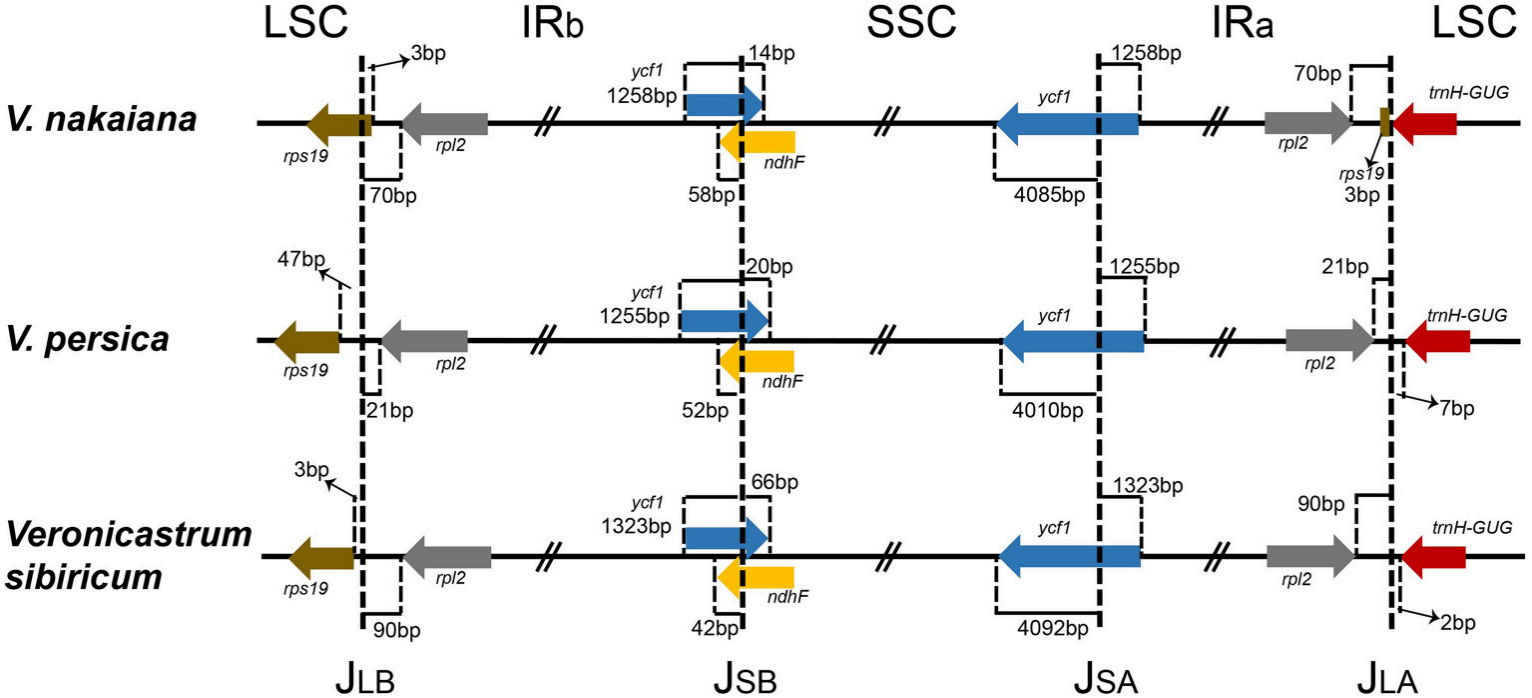
**Comparison of the LSC, IR, and SSC junction positions among three Veroniceae chloroplast genomes**.

The IR/LSC and IR/SSC borders of the Veroniceae cp genomes were compared (Figure 2). In the *V. nakaiana* chloroplast genome, the IR extended into the *rps19* gene, resulting in a short *rps19* pseudogene of 3 bp at the IR/LSC border. However, the *rps19* gene of *V. persica* and *V. sibiricum* was separated from the LSC/IRb border by 3–47 bp and not duplicated in the IRa/LSC border. The position of *ycf1* in the IR regions varied from 1255 to 1323 bp. The *ndhF* gene and *ycf1* gene overlapped in the IRb/SSC border (72 bp in *V. nakaiana*, 72 bp in *V. persica*, and 108 bp in *V. sibiricum*). The *trnH* gene of the three Veroniceae species was located in the LSC region (separated from the IR/LSC border by 0–7 bp).

### SSR and repeat analysis

**Figure 3:**
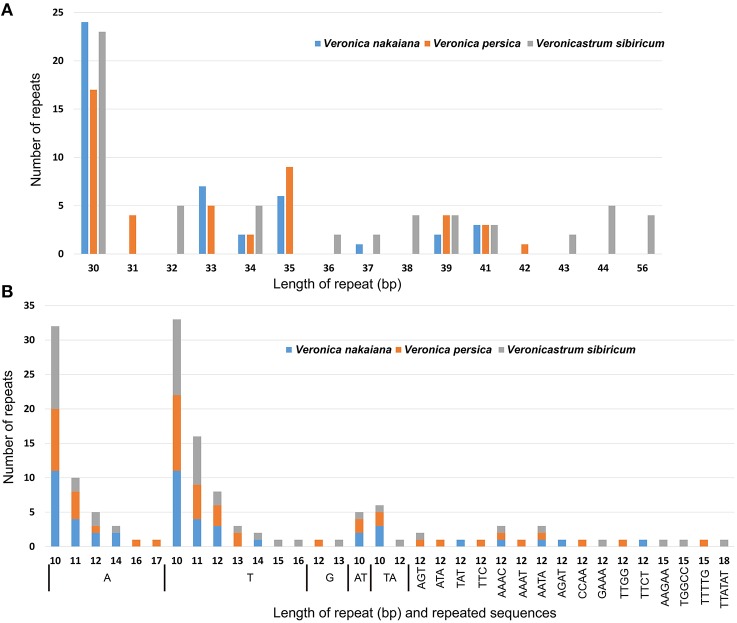
**Analyses of repeated sequences in the three Veroniceae chloroplast genomes**. **(A)** Frequency of repeat sequences determined by REPuter. **(B)** Frequency of simple sequence repeats (SSRs) by Phobos.

We used REPuter to analyze the repeat sequence of three Veroniceae cp genomes and found forward repeats, palindrome repeats and reverse repeats of at least 30 bp long per repeat unit with a sequence identity of ≥90% (Figure 3). *V. nakaiana* contained 15 forward repeats, 18 palindrome repeats, and 2 reverse repeats (Supplementary Table 2). Overall, 33 repeats were 30–40 bp long, with two repeats 41 long. *V. persica* contained 16 forward repeats and 19 palindrome repeats. Of these, 32 repeats were 30–40 bp long, two repeats were 41 bp long, and one repeat was 42 bp long. *V. sibiricum* contained 22 forward repeats and 21 palindrome repeats. Thirty-two repeats were 30–40 bp long, seven repeats were 40–50 bp long, and four repeats were 56 bp long (Supplementary Table 2). Most of these repeats had lengths between 30 and 41 bp. A total of 113 repeats were detected in these cp genomes, 46% (53 repeats) of which were direct repeats, 51% (58 repeats) were palindromic repeats, and 3% (2 repeats) reverse repeats. The five repeats shared among *V. nakaiana, V. persica*, and *V. sibiricum* were as follows: a 30-bp sequence in the rrn4.5-rrn5 spacer, *psaB, psaA, ycf2*, and a 41 bp repeat that occurred in the *rps12*-*trnV*-*GAC* intergenic spacer. Four repeats were only shared between *V. nakaiana* and *V. sibiricum*: a 30 bp direct repeat in the *psbI*-*trnS*-*GCU* intergenic spacer and *trnS*-*GGA* tRNA, and a 30 bp palindrome repeat in the *accD*-*psaI* intergenic spacer and *rpl16* intron.

SSRs are repeated DNA sequences consisting of tandem repeats 1–10 bp in length per unit distributed throughout the genome (Figure 3B). SSRs are highly polymorphic and therefore useful as molecular markers and for population genetics (Powell et al., 1995; Zhang et al., 2012). We detected SSRs longer than 10 bp in three chloroplast genomes. Additionally, the total number of SSRs was 48 in *V. nakaiana*, 51 in *V. persica*, and 53 in *V. sibiricum* (Supplementary Table 3). The majority of SSRs in all species are A/T mononucleotides. Chloroplast genome SSRs were composed of adenine or thymine repeats, and rarely contained tandem guanine (G) or cytosine (C) repeats (Kuamg et al., 2011; Qian et al., 2013). Most SSRs were located in intergenic regions (69%), whereas 14 and 17% were located in introns and genes, respectively. Overall, 25 SSRs were located in the genes in three Veroniceae. Among these, three (*rpoA, rpoC2, ndhD*) were shared by all three Veroniceae species.

### Phylogenetic analyses of 78 coding genes in chloroplast genome

Phylogenetic analysis of coding genes was not resolved in previous studies of Lamiales plants. Previous phylogenetic studies of Veroniceae have focused on non-coding regions such as rapidly evolving chloroplast DNA (Rahmanzadeh et al., 2005; Schäferhoff et al., 2010). Our analyses of the extended data with much wider coding genes in Lamiales support a monophyletic family. The monophyly of the Lamiales families, its circumscription is clearly revealed. The position of Veroniceae within the Plantaginaceae corresponded with that reported by Schäferhoff et al. (2010).

**Figure 4:**
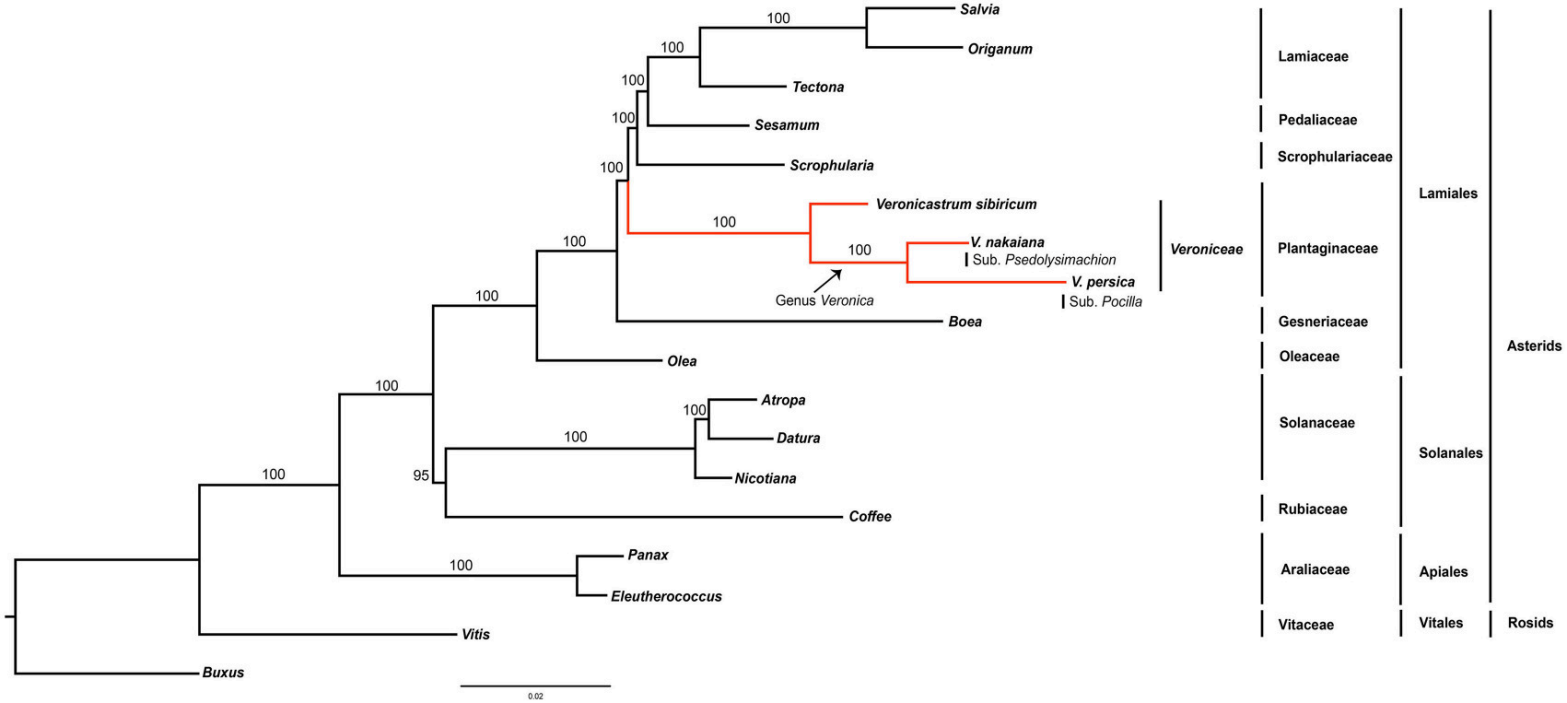
**Phylogenetic tree reconstruction of 18 taxa using maximum likelihood based on concatenated sequences of 78 protein coding genes**. Bootstrap support values >50% are given at the nodes.

In this study, maximum likelihood phylogenetic analysis of 78 protein coding genes was conducted and 18 taxa were aligned (Figure 4). This alignment was used for analyses of 71,314 bp. The Asterids clade was monophyletic and well supported by the bootstrap value (100%). Within the order, two well-supported clades, Lamiales and Solanales, were distinguished. Veroniceae was highly supported (100%) and a sister to Scrophulariaceae, Pedaliaceae, and Lamiaceae.

### Divergence sequence hotspots in veroniceae

The coding genes, non-coding regions and intron regions were compared among the three Veroniceae species divergence hotspots. We generated more 200 bp of 114 regions (coding genes, non-coding regions and intron regions) from three Veroniceaea species and the nucleotide variability (Pi) values calculated with the DnaSP 5.0 software.

**Figure 5:**
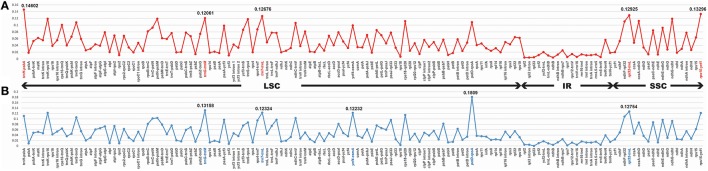
**Comparison of the nucleotide variability (Pi) values in Veroniceae. (A)** *Veronica nakaiana* vs. *V. persica* vs. *Veronicastrum sibiricum*, **(B)** *Veronica nakaiana* vs. *V. persica.*

Among Veroniceae three species (*V. nakaiana, V. persica*, and *V. sibiricum*) values ranged from 0.00403 (*rpl2* gene) to 0.14602 (*trnH*-*psbA* region). The IR region is much more conserved than the LSC and SSC regions. Five of these, *trnH*-*psbA, trnG*-*trnM, trnT*-*trnL, rpl32*-*trnL*, and *rps15*-*ycf1*, showed high levels of variation (Table 2). Three of these (*trnH-psabA, trnG-trnM, trnT-trnL*) were located in the LSC and two region (*rpl32-trnL* and *rps15-ycf1*) were in the SSC region (Figure 5A).

**Table 2:** **Five regions of highly variable sequences of tribe Veroniceae and genus *Veronica***.

		**Nucleotide diversity (Pi)**	**Total number of mutation (Eta)**
Five variable regions in tribe Veroniceae^*^	*trnH*-*psbA*	0.14602	51
	*trnG*-*trnM*	0.12061	28
	*trnT*-*trnL*	0.12676	111
	*rpl32*-*trnL*	0.12925	146
	*rps15*-*ycf1*	0.13296	73
Five variable regions in genus Veronica^**^	*trnG*-*trnM*	0.13158	20
	*trnT*-*trnL*	0.12324	70
	*ycf4*-*cemA*	0.12232	80
	*petD*-*rpoA*	0.18090	36
	*rpl32*-*trnL*	0.12754	94

* Veronicastrum sibiricum (Genus Veronicastrum) vs. Veronica nakaiana (Genus Veronica subgenus Pseudolysimahium) vs. V. persica (Genus Veronica subgenus Pocilla).

** Veronica nakaiana (Genus Veronica subgenus Pseudolysimahium) vs. V. persica (Genus Veronica subgenus Pocilla).

The sequence divergence between subgenus *Pseudolysimachium* and subgenus *Pocilla* varied from 0 (*rpl23* gene) to 0.1809 (*petD*-*rpoA* region), and most of the variable loci were in the LSC regions (four in the LSC region and one in the SSC region). Five regions (*trnG*-*trnM, trnT*-*trnL, ycf4*-*cemA, petD*-*rpoA*, and *rpl32-trnL*) were highly informative phylogenetic markers (Figure 5B, Table 2).

## Discussion

### The chloroplast genome of veroniceae

Recently, some taxonomic studies have used the chloroplast genome to evaluate the relationship of related species. For example, the chloroplast genome of two species of *Machilus* (Song et al., 2015) and four species of *Tilia* (Cai et al., 2015) were utilized to evaluate the phylogenomics and relationship within the family. Comparative analyses of three members of the Veroniceae chloroplast genome also showed highly conserved structures and genes. The size of *V. nakaiana, V. persica*, and *V. sibiricum* ranged from 150,198 to 152,930 bp. Three Veroniceae contained the same coding genes, tRNAs and rRNAs. However, the start codon of the *rps19* gene in *V. sibiricum* was changed to GTG. Similar findings have been detected in *Cymbidium* (Yang et al., 2013).

Land plants have a highly conserved chloroplast genome, but four junctions with changed genome sizes (Plunkett and Downie, 2000; Hansen et al., 2007; Qian et al., 2013). For example, Geraniaceae (Blazier et al., 2011) was found to lack a copy of the IR region, *Tetracentron* (Sun et al., 2013) showed expansion/contraction of the IR region, subfamily Apioideae (Plunkett and Downie, 2000) showed variations in J_LB_ (LSC/IRb region) and Elaeagnaceae contained *trnH* gene duplication in the IR region (Choi et al., 2015).

We compared three Veroniceae species, among which *V. nakaiana* was unique in that its *rps19* gene was duplicated and J_LB_ (LSC/IRb) and J_LA_ (LSC/IRa) differences occurred (Figure 2). The *rpl2* genes of *V. nakaiana* and *V. sibiricum* were separated by >50 bp at the J_LB_, whereas that in *V. persica* was separated by 21 bp. Three types of J_LB_ based on expansion/contraction have been characterized in Veroniceae.

### Divergence region of veroniceae plastid genome

The ITS region and cpDNA non-coding regions have been widely used to investigate taxonomy and molecular phylogeny at the interspecific level (Taberlet et al., 1991; Baldwin, 1992). Recently, studies of the chloroplast genome showed divergent sequences in plants (Parks et al., 2009; Yang et al., 2013). In Veroniceae, Albach (Albach and Chase, 2001, 2004; Albach et al., 2004a,b,c; Albach and Meudt, 2010) tested the ITS and *CYC2* of nuclear ribosomal DNA and some chloroplast DNA (*trnL*-*F, trnL, rps16, rpoB*-*trnC*, and *trnH*-*psbA*). However, the relationships among the 12 genera in *Veronica* are not well supported.

Based on our study, the largest sequence divergence regions were *trnH*-*psbA, trnG*-*trnM, trnT*-*trnL, rpl23*-*trnL*, and *rps15*-*ycf1* in Veroniceae plants (*V. nakaiana, V. persica*, and *V. sibiricum*). In Veronica, *trnG*-*trnM, trnT*-*trnL, ycf4*-*cemA, petD*-*rpoA*, and *rpl2*-*trnL* was a highly variable locus (Figure 5B). The total sequence divergence hotspot regions in Veroniceae and *Veronica* were *trnH-psbA, trnG-trnM, trnT-trnL, rpl32-trnL, rps15-ycf1, ycf4-cemA, petD-rpoA*, and *rpl32-trnL* (Table 2).

Here, we showed the eight DNA variable regions that could be used as molecular markers in Veroniceae and *Veronica* (Figure 5, Table 2 and Supplementary Table 4). The results presented here will be helpful to systematics of Veroniceae and evaluating the relationships among the 12 subgenera of *Veronica*.

## Author contributions

Conceived and designed the experiments: SP, KC, MC. Performed the experiments: KC, SP. Analyzed the data: KC, MC. Wrote the paper: KC.

### Conflict of interest statement

The authors declare that the research was conducted in the absence of any commercial or financial relationships that could be construed as a potential conflict of interest.
