# Supplementary material for: The Complete Chloroplast Genome Sequences of Three Veroniceae Species (Plantaginaceae): Comparative Analysis and Highly Divergent Regions
Source: Front Plant Sci. 2016 Mar 23;7:355. doi: 10.3389/fpls.2016.00355 (PMC4804161; doi:10.3389/fpls.2016.00355)
Supplement: Supplementary file 1 [file Table1.DOCX]

| **Supplementary material 1.** Phylogenetic analyses with GenBank accession numbers | | |
| --- | --- | --- |
| **A.** 78 coding genes phylogenetic tree | |  |
| Taxon | Genbank Accession number |  |
| *Buxus microphylla* | NC_016468 |  |
| *Vitis vinifera* | NC_007957 |  |
| *Eleutherococcus senticosus* | NC_016430 |  |
| *Panax ginseng* | KF_431956 |  |
| *Coffea arabica* | EF044213 |  |
| *Nicoiana tabacum* | NC_001879 |  |
| *Datura stramonium* | NC_018117 |  |
| *Atropa belladonna* | AJ316582 |  |
| *Olea europaea* | NC_013707 |  |
| *Boea hygrometrica* | NC_016468 |  |
| *Scrophularia takesimensis* | NC_026202 |  |
| *Sesamum indicum* | JN637766 |  |
| *Tectona grandis* | NC_020098 |  |
| *Origanum vulgare* | JX880022 |  |
| *Salvia miltiorrhiza* | NC_020431 |  |
| *Veronica nakaiana* | KT633216 |  |
| *Veronica persica* | KT724052 |  |
| *Veronicastrum sibiricum* | KT724053 |  |
