# Supplementary material for: The Complete Chloroplast Genome Sequences of Three Veroniceae Species (Plantaginaceae): Comparative Analysis and Highly Divergent Regions
Source: Front Plant Sci. 2016 Mar 23;7:355. doi: 10.3389/fpls.2016.00355 (PMC4804161; doi:10.3389/fpls.2016.00355)
Supplement: Supplementary file 2 [file Table2.DOCX]

**Supplementary material 2.** Repeat sequence of three Veroniceae species

The repeat sequences in the *Veronica nakaiana* chloroplast genome.

| Repeat  Size | Type | Start position of 1^st^ repeat | Start position the repeat found in other region | Location | Region |
| --- | --- | --- | --- | --- | --- |
| 30 | P | 7862 | 44820 | IGS(*psb*I-*trn*S-GCU), *trn*S-GGA | LSC |
| 30 | F, P | 106024 | 106055, 129429, 129460 | IGS(rrn4.5-rrn5) | IR |
| 30 | F | 41512 | 41543 | IGS(*psa*A-*ycf*3) | LSC |
| 30 | R | 46970 | 46370 | Intron(*trn*L-UAA) | LSC |
| 30 | P | 114003 | 114003 | IGS(*ccs*A-ndhD) | SSC |
| 30 | P | 34920 | 44820 | IGS(*psb*C-*trn*S-UGA), *trn*S-GCA | LSC |
| 30 | P | 38061 | 40285 | *psa*B, *psa*A | LSC |
| 30 | P | 58574 | 81244 | IGS(*acc*D-*psa*I), Intron(*rpl*16) | LSC |
| 30 | P | 60200 | 60320 | IGS(*ycf*4-*cem*A) | LSC |
| 30 | F, P | 87973 | 88015, 147469, 147511 | *ycf*2 | IR |
| 33 | P | 56122 | 56122 | IGS(*rbc*L-*acc*D) | LSC |
| 33 | F, P | 90380 | 90398, 145083, 145101 | *ycf*2 | IR |
| 33 | P | 111654 | 11654 | IGS(*ndh*F-*rpl*32) | SSC |
| 34 | F | 7855 | 34913 | IGS(*psb*I-*trn*S-GCU) | LSC |
| 35 | F, P | 90383 | 90401, 90419, 145060, 145078, 145096 | *ycf*2 | IR |
| 37 | R | 4347 | 4347 | IGS(*trn*K-UUU-*rps*16) | LSC |
| 39 | F | 43095 | 118952 | Intron(*ycf*3) | LSC |
| 41 | F, P | 97394 | 118950, 138079 | Intron(*rps*12-*trn*V-GAC), Intron(*ndh*A) | IR,SSC |

*F: Forward, P-palindrome, R-reverse

| Repeat  Size | Type | Start position of 1^st^ repeat | Start position the repeat found in other region | Location | Region |
| --- | --- | --- | --- | --- | --- |
| 30 | P | 235 | 279 | IGS(*trn*H-GUG-*psb*A) | LSC |
| 30 | F, P | 104448 | 104479, 127539, 127570 | IGS(rrn4.5-rrn5) | IR |
| 30 | F | 37482 | 39706 | *psaB*, psaA | LSC |
| 30 | F, P | 42574 | 95849, 136169 | Intron(*ycf3*), IGS(*rps*12-*trn*V-GAC) | LSC, IR |
| 30 | P | 49646 | 49722 | IGS(*ndh*C-*trn*V-UAC) | LSC |
| 30 | F, P | 86459 | 86501, 145517, 145559 | *ycf2* | IR |
| 31 | F | 7736 | 34366 | IGS(*psb*I-*trn*S-GCU) | LSC |
| 31 | P | 41904 | 41904 | Intron(*ycf3*) | LSC |
| 33 | P | 55409 | 55409 | IGS(*rbc*L-*acc*D) | LSC |
| 33 | F, P | 88869 | 88887, 143128, 143146 | *ycf2* | IR |
| 34 | P | 7736 | 44281 | IGS(*psb*I-*trn*S-GCU), IGS(*trn*S-GGA-*rps*4) | LSC |
| 35 | F, P | 88872 | 88908, 88890, 143105, 143123, 143141 | *ycf*2 | IR |
| 35 | F | 143105 | 143123, 143141 | *ycf*2 | IR |
| 39 | F, P | 42562 | 95837, 117209, 136172 | Intron(*ycf*3), IGS(*rps*12-*trn*V-GAC),Intron(*ndh*A) | LSC,IR,SSC |
| 41 | F, P | 95835 | 117207, 136172 | IGS(*rps*12-*trn*V-GAC) | IR |
| 42 | P | 63703 | 63703 | IGS(*psb*E-*pet*L) | LSC |

The repeat sequences in the *Veronica persica* chloroplast genome.

*F: Forward, P-palindrome, R-reverse

| Repeat  Size | Type | Start position of 1^st^ repeat | Start position the repeat found in other region | Location | Region |
| --- | --- | --- | --- | --- | --- |
| 30 | F | 8027 | 44969 | IGS(*psb*I-*trn*S-GCU) , *trn*S-GGA | LSC |
| 30 | F, P | 106431 | 106431, 106462, 130054, 130085 | IGS(rrn4.5-rrn5) | IR |
| 30 | F | 9553 | 35963 | IGS(*trn*G-UCC-*trn*R-UCU), IGS(*trn*G-GCC-*trn*M-CAU) | LSC |
| 30 | P | 35003 | 44969 | IGS(*psb*C-*trn*S-UGA), *trn*S-GGA | LSC |
| 30 | F | 38184 | 40408 | *psa*B, *psa*A | LSC |
| 30 | P | 58997 | 81611 | IGS(*acc*D-psaI), Intron(*rpl*16) | LSC |
| 30 | F, P | 88404 | 88446, 148070, 148112 | *ycf2* | IR |
| 30 | F | 145692 | 145710 | *ycf2* | IR |
| 30 | F | 148070 | 148112 | *ycf2* | IR |
| 32 | P | 30855 | 30855 | IGS(*trn*E-UUC-*trn*T-GGU) | LSC |
| 32 | F | 8025 | 35001 | IGS(*psb*I-*trn*S-GCU), IGS(*psb*C-*trn*S-UGA) | LSC |
| 32 | F | 73649 | 73650 | IGS(*psb*B-*psb*T) | LSC |
| 34 | F, P | 90837 | 90855, 145657, 145693, 145675 | *ycf2* | IR |
| 36 | F | 83175 | 83214 | *rpl22* | LSC |
| 37 | F | 145684 | 145702 | *ycf2* | IR |
| 38 | F, P | 90811 | 90847, 145661, 145697 | *ycf2* | IR |
| 39 | F, P | 43214 | 97808, 119519, 138699 | Intron (*clpP*), IGS(*rps*12-*trnV*-GAC) | LSC, IR |
| 41 | F, P | 97806 | 119517, 138699 | IGS(*rps*12-*trn*V-GAC) | IR |
| 43 | F | 50621 | 50646 | IGS(*ndh*C-*trn*V-UAC) | LSC |
| 44 | F | 73943 | 73943 | IGS(*psb*T-*psb*N) | LSC |
| 44 | F, P | 90827 | 90845, 145657, 145675 | *ycf2* | IR |
| 56 | F, P | 90811 | 90829, 145661, 145679 | *ycf2* | IR |

The repeat sequences in the *Veronicastrum sibiricum* chloroplast genome.

*F: Forward, P-palindrome, R-reverse
