# Supplementary material for: The Complete Chloroplast Genome Sequences of Three Veroniceae Species (Plantaginaceae): Comparative Analysis and Highly Divergent Regions
Source: Front Plant Sci. 2016 Mar 23;7:355. doi: 10.3389/fpls.2016.00355 (PMC4804161; doi:10.3389/fpls.2016.00355)
Supplement: Supplementary file 3 [file Table3.DOCX]

**Supplementary material 3.** Simple sequence repeats of three Veroniceae species

Simple sequence repeats in the *Veronica nakaiana* chloroplast genome.

| Repeat unit | Length (bp) | Number of SSRs | Start position |
| --- | --- | --- | --- |
| A | 10 | 11(8/3/0) | IGS(trnK-UUU-rps16), IGS(psbM-trnD-GUC), Intron(ycf3), IGS(trnS-GGA-rps4), Intron(trnL-UAA), IGS(atpB-rbcL), IGS(rps18-rpl20), IGS(rpl20-rps12), Intron(clpP), IGS(psbB-psbT), IGS(rps12-rps7) |
|  | 11 | 4(3/1/0) | Intron(atpF), IGS(trnT-GGU-psbD), IGS(ycf3-trnS-GGA), IGS(accD-psaI) |
|  | 12 | 2(2/0/0) | IGS(psbZ-trnG-GCC), IGS(ycf4-trnL-UAA) |
|  | 14 | 2(2/0/0) | IGS(atpH-atpI), IGS(rps4-trnL-UAA) |
| T | 10 | 11(9/0/2) | IGS(trnT-GGU-psbD), IGS(trnT-GGU-psbD), IGS(trnF-GAA-ndhJ), atpB, IGS(psbE-petL), IGS(petG-trnW-CCA), IGS(psbB-psbT), rpoA, IGS(rpoA-rps11), IGS(rps7-rps12), IGS(rpl32-trnL-UAG) |
|  | 11 | 4(3/0/1) | IGS(atpG-atpI), rpoC2, IGS(trnM-CAU-atpE), IGS(psaI-ycf4) |
|  | 12 | 3(2/1/0) | IGS(trnC-trnV-UAC), IGS(ycf4-cemA), Intron(petB) |
|  | 14 | 1(0/0/1) | ycf1 |
| AT | 10 | 2(1/0/1) | rpoC2, IGS(psaI-ycf4) |
| TA | 10 | 3(2/0/1) | IGS(trnK-UUU-rps16), IGS(trnS-GCU-trnG-UCC), rps19 |
| TAT | 12 | 1(1/0/0) | IGS(trnS-GCU-rnG-UCC) |
| AATA | 12 | 1(0/0/1) | ndhD |
| AGAT | 12 | 1(1/0/0) | IGS(petD-rpoA) |
| AAAC | 12 | 1(1/0/0) | IGS(rpl33-rps16) |
| TTCT | 12 | 1(0/1/0) | Intron(trnG-UCC) |

*(IGS/Intron/Gene)

| Repeat unit | Length (bp) | Number of SSRs | Start position |
| --- | --- | --- | --- |
| A | 10 | 9 (8/1/0) | IGS(trnT-GGU-psbD), IGS(psbZ-trnG-GCC), IGS(psaA-ycf3), Intron(ycf3),IGS(petA-psbJ), IGS(petD-rpoA), IGS(rpl14-rpl16), IGS(ndhB-rps7), IGS(ndhG-ndhI) |
|  | 11 | 4(2/2/0) | Intron(trnL-UAA), IGS(atpB-rbcL), Intron(rpl16), IGS(trnN-GUU-trnR-ACG) |
|  | 12 | 1(0/1/0) | Intron(trnG-UCC) |
|  | 16 | 1(1/0/0) | IGS(ndhD-psaC) |
|  | 17 | 1(1/0/0) | IGS(rpl32-trnL-UAG) |
| T | 10 | 11(5/2/4) | IGS(psbK-psbI), Intron(atpF), IGS(atpH-atpI), rpoC2(2), Intron(trnL-UAA), IGS(trnF-GAA-ndhJ), IGS(petA-psbJ), rpoA, ycf1, IGS(rps7-ndhB) |
|  | 11 | 5(3/0/2) | rpoC2, IGS(trnM-CAU-atpE), IGS(rbcL-accD), IGS(trnR-ACG-trnN-GUU), ycf2 |
|  | 12 | 3(3/0/0) | IGS(rps4-trnT-UGU), IGS(trnL-UAA-trnF-GAA), IGS(trnF-GAA-ndhJ) |
|  | 13 | 2(2/0/0) | IGS(psaI-ycf4), IGS(ndhF-rpl32) |
| G | 12 | 1(1/0/0) | IGS(trnC-GCA-petN) |
| AT | 10 | 2(1/0/1) | rpoC2, IGS(ndhK-ndhC) |
| TA | 10 | 2(0/1/1) | Intron(rps16), rps19 |
| AGT | 12 | 1(1/0/0) | IGS(rps4-trnT-UGU) |
| ATA | 12 | 1(1/0/0) | IGS(rps16-trnQ-UUG) |
| TTC | 12 | 1(0/0/1) | psbC |
| AAAC | 12 | 1(1/0/0) | IGS(rpl33-rps18) |
| AAAT | 12 | 1(1/0/0) | IGS(ndhG-ndhI) |
| AATA | 12 | 1(0/0/1) | ndhD |
| TTGG | 12 | 1(1/0/0) | IGS(trnR-ACG-trnN-GUU) |
| CCAA | 12 | 1(1/0/0) | IGS(trnN-GUU-trnR-ACG) |
| TTTTG | 15 | 1(0/0/1) | ycf1 |

Simple sequence repeats in the *Veronica persica* chloroplast genome.

Simple sequence repeats in the *Veronicastrum sibiricum* chloroplast genome.

| Repeat unit | Length (bp) | Number of SSRs | Start position |
| --- | --- | --- | --- |
| A | 10 | 12(11/1/0) | IGS(atpG-atpI), IGS(rps2-rpoC2), IGS(psbZ-trnG-GCC), IGS(ycf3-trnS-GGA), IGS(atpB-rbcL), IGS(petA-psbJ)(2), IGS(psaJ-rpl33), IGS(rps18-rpl20), Intron(clpP), IGS(ndhB-rps7), IGS(rpl32-trnL-UAG) |
|  | 11 | 2(2/0/0) | IGS(trnT-UGU-trnL-UAA), IGS(accD-psaI) |
|  | 12 | 2(1/1/0) | IGS(psbM-trnD-GUC), Intron(rpl16) |
|  | 14 | 1(1/0/0) | IGS(ndhF-rpl32) |
| T | 10 | 11(5/2/4) | Intron(rps16), Intron(atpF), IGS(ndhC-trnV-UAC)(2), atpB, IGS(rbcL-accD), clpP, rpoA, ndhF, IGS(rpl32-trnL-UAG), IGS(rps7-ndhB) |
|  | 11 | 7(3/3/1) | Intron(rps16), IGS(atpA-atpF), rpoC2, Intron(ycf3), Intron(rps4-trnT-UGU), IGS(ndhF-rpl32), IGS(rpl32-trnL-UAG) |
|  | 12 | 2(1/1/0) | IGS(psbC-trnS-UGA), Intron(petB) |
|  | 13 | 1(1/0/0) | IGS(rps2-rpoC2) |
|  | 14 | 2(2/0/0) | IGS(trnT-UGU-trnL-UAA), IGS(trnF-GAA-ndhJ) |
|  | 15 | 1(1/0/0) | IGS(psbB-psbT) |
|  | 16 | 1(1/0/0) | IGS(rps18-rpl20) |
| G | 13 | 1(1/0/0) | IGS(psbB-psbT) |
| AT | 10 | 1(0/0/1) | rpoC2 |
| TA | 10 | 1(1/0/0) | IGS(rps15-ycf1) |
|  | 12 | 1(1/0/0) | IGS(trnS-GCU-trnG-UCC) |
| AGT | 12 | 1(1/0/0) | IGS(rps4-trnT-UGU) |
| AAAC | 12 | 1(1/0/0) | IGS(rpl33-rps18) |
| AATA | 12 | 1(0/0/1) | ndhD |
| GAAA | 12 | 1(1/0/0) | IGS(ycf4-cemA) |
| AAGAA | 15 | 1(0/1/0) | Intron(petB) |
| TGGCC | 15 | 1(1/0/0) | IGS(trnS-GCU-trnG-UCC) |
| TTATAT | 18 | 1(1/0/0) | IGS(trnL-UAG-ccsA) |
